# Membrane Protein Crystallisation: Current Trends and Future Perspectives

**DOI:** 10.1007/978-3-319-35072-1_5

**Published:** 2016-04-28

**Authors:** Joanne L. Parker, Simon Newstead

**Affiliations:** Department of Biochemistry, University of Oxford, Oxford, OX1 3QU UK

**Keywords:** Membrane protein, Crystallisation, Screen development, Detergent selection

## Abstract

Alpha helical membrane proteins are the targets for many pharmaceutical drugs and play important roles in physiology and disease processes. In recent years, substantial progress has been made in determining their atomic structure using X-ray crystallography. However, a major bottleneck still remains; the identification of conditions that give crystals that are suitable for structure determination. Over the past 10 years we have been analysing the crystallisation conditions reported for alpha helical membrane proteins with the aim to facilitate a rational approach to the design and implementation of successful crystallisation screens. The result has been the development of MemGold, MemGold2 and the additive screen MemAdvantage. The associated analysis, summarised and updated in this chapter, has revealed a number of surprisingly successfully strategies for crystallisation and detergent selection.

## Introduction

Protein crystallisation is often described as a ‘black box’ process, full of mystery and superstition. In fact crystallisation itself is a well documented process following well understood physical chemistry laws and involving the supersaturation of the protein of interest to coax the molecules into a regular three-dimensional crystal (McPherson and Gavira 2014; Chayen and Saridakis 2008). Although not the topic of this chapter, many excellent sources of information are available on how to set up these conditions, using either vapor diffusion (Delmar et al. 2015), microbatch (Chayen 1998) or free-interface diffusion (Segelke 2005). The mystery begins when we try to consider which conditions will coax the proteins to assemble in a regular form and produce diffraction quality crystals. For many years, the standard experimental set up has involved screening your purified, homogenous protein sample against commercial, sparse-matrix style ‘Crystallisation screens’ (Luft et al. 2014). The idea behind these screens was to sample as much ‘crystallisation space’ as possible with the minimal protein amount (Newman et al. 2007). Of course, many of these commercial screens were based on the currently available information regarding soluble protein Crystallisation. This included the success of large molecular weight (MW) polymers, particularly polyethylene glycol (PEG). High concentrations of salt were also used, as these would naturally help to ‘salt out’ the protein and hopefully grow protein crystals (Page and Stevens 2004).

Membrane proteins however are different. The requirement to extract these proteins from the membrane using Detergents, whilst simultaneously keeping them folded and stable in solution creates a new set of unknown variables (Iwata 2003). Added to this the fact that in a crystallisation experiment involving Detergents solubilised membrane proteins, the actual entity being crystallised is the Detergents-protein complex and not simply the protein alone (Kunji et al. 2008; Bill et al. 2011). It was against this backdrop that in 2008 a comprehensive analysis of membrane protein crystallization conditions was published (Newstead et al. 2008a). The idea behind this analysis was simple, the number of crystal structures had just reached 121 and our aim was to analyse these conditions and draw conclusions as to which chemicals were successful in growing membrane protein crystals. Could any trends be observed and could this information be used to improve success rates in current projects? The result of this analysis was the release of the first rationally designed sparse matrix style membrane protein Crystallisation screen, MemGold (Newstead et al. 2008a).

In the following years the pace of membrane protein structure determination has increased exponentially (White 2007). This increase is due to progress being made in tackling many of the hurdles faced in determining the crystal structure of membrane proteins (Bill et al. 2011; Ghosh et al. 2015). This includes advances in protein production using recombinant systems (Tate et al. 2003; Chen et al. 2013), methods for screening stability (Drew et al. 2008; Kawate and Gouaux 2006; Sonoda et al. 2011) and in X-ray data collection using microfocus beamlines, fast read out detectors and modifications to sample application (Nogly et al. 2005). More recent progress has been made in protein engineering, resulting in either increases in protein stability (Tate and Schertler 2009) or the introduction of additional Crystallisation scaffolds, such as T4 lysozyme or BRIL (Chun et al. 2012). However, growing well-ordered three-dimensional crystals still represents a significant hurdle. In 2012, we followed up our first analysis with another review of the current trends in Crystallisation, this time based on 254 examples from the Protein Data Bank (PDB) (Parker and Newstead 2012). Our results showed that the initial trends described in 2008 had broadly held, but revealed intriguing new developments such as an increase in the number of cases where additional or mixed Detergents had been required and changes in the types of membrane protein being crystallised. The new information enabled the development a sister crystallization screen, MemGold 2, to complement the original MemGold screen released 4 years earlier. In addition to our analysis of crystallisation conditions, an in depth analysis of additives was now possible. The use of additional chemicals to optimise initial crystals to improve diffraction quality is well documented and many commercial kits are available (Chayen and Saridakis 2008; Cudney et al. 1994). An additive screen targeted specifically for membrane proteins however, had so far remained absent from the commercial market. A specific membrane protein additive screen was therefore suggested to facilitate crystal Crystal optimisation and released along with MemGold 2, called MemAdvantage.

As of August 2015, the number of Crystallisation examples in our database is more than 500 and in this chapter we present an updated analysis from these conditions. Here we compare the results of these past analyses with each other and with those focused on soluble proteins (Fazio et al. 2015). The aim of this chapter is to equip the protein crystallographer with the knowledge to design their own screens using information that is up to date and relevant to membrane protein samples.

## Current Trends in the Number and Types of Alpha helical Membrane Protein Structures

**Fig. 5.1:**
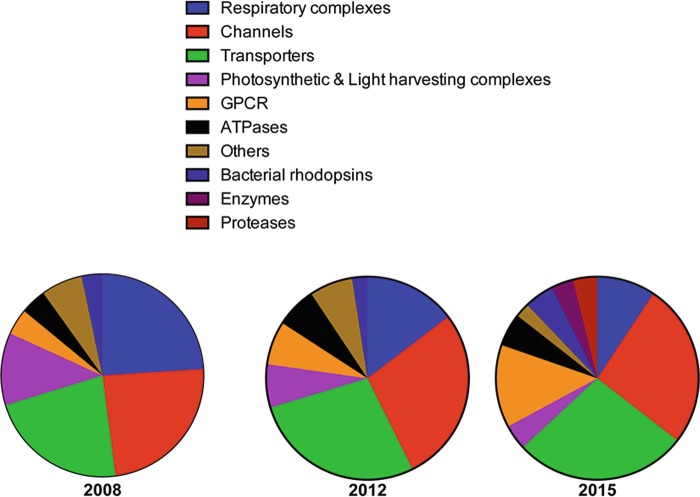
Number and types of Alpha helical membrane protein structures reported from 2008 to present (2015). Pie charts showing the change in the proportion of structures belonging to each family group from 2008, 2012 and 2015. Membrane proteins were broken down into the following families: Respiratory complexes (*blue*), G protein coupled receptors (GPCRs) (*orange*), ATPases (*black*), Bacterial Rhodopsins (*dark blue*), Enzymes (*purple*), Proteases (*dark red*) and where a protein didn’t fit in these categories, others (*brown*)

Since 2008, an additional 448 novel Alpha helical membrane protein (MP) structures have been added to our original Crystallisation database, bringing the total number of entries to 569. We have previously grouped these into eight different families, broadly divided by function (Fig. 5.1). However, we noticed a significant growth in the number of enzymes being reported. Therefore, we have included two additional families for proteases and other enzymes, bringing the total family count up to 10. The ‘Other’ family now contains examples of either single members of a functional family, such as the tight junction Claudin-15 (PDB:4P79) and BcsA-BcsB cellulose synthease (PDB:4HG6). The data clearly show an increase in the determination of channel and transporter structures, from 29 and 27 to 149 and 157, respectively. This has occurred with a continued decrease in the overall percentage of respiratory complexes, from 24 % to 9 %. Possibly the largest change from 2012 has been in the G protein coupled receptor (G protein coupled receptors (GPCRs))G protein coupled receptors (GPCRs) family, which now makes up 13 % of the database with >70 structures, up from 17 structures in 2012. Significant progress has been made in the structure determination of G protein coupled receptors (GPCRs) due to a number of technological advancements in protein engineering and lipidic mesophase Lipidic mesophase crystallisation (LCP) (LCP) (Ghosh et al. 2015); we discuss LCP Crystallisation in more detail towards the end of the chapter. The contribution from the photosynthetic and light harvesting complexes (LHCs) has also reduced, falling from 7 % in 2012 to 4 % in 2015. The number of ATPase structures has doubled from 16 to 30 since 2012, but their total share of the database has remained the same, at 6 %.

## Detergents Selection

**Fig. 5.2:**
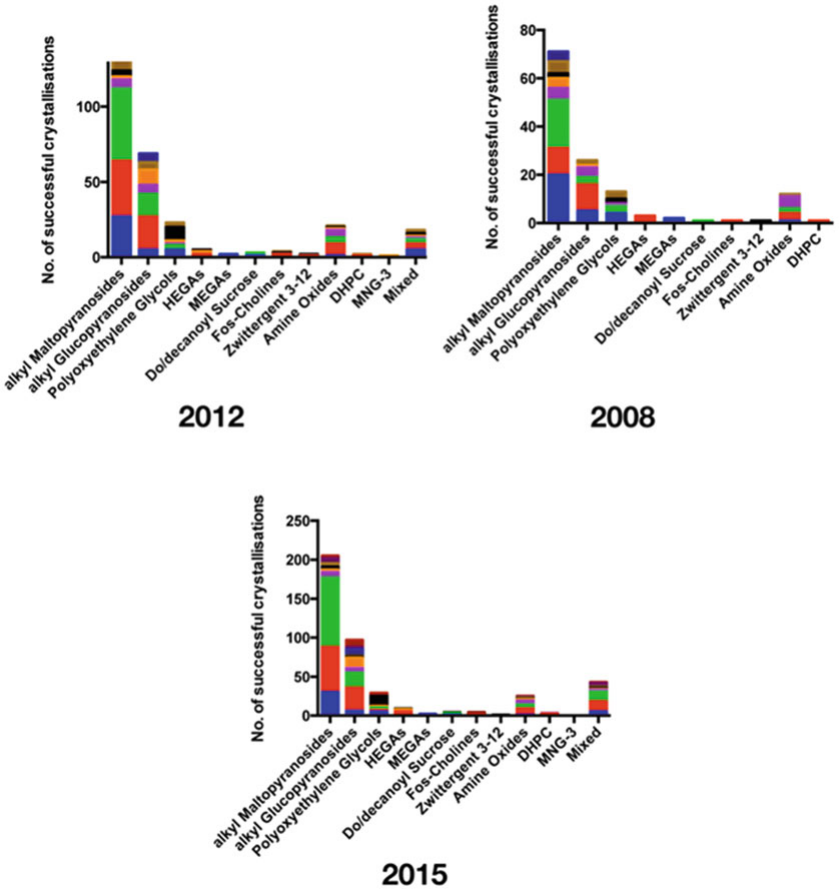
Analysis of successful CrystallisationDetergents used for Alpha helical membrane proteins. Numbers for each Detergents class are shown and the bars are subdivided to represent the different membrane protein families. Respiratory complexes (*blue*), G protein coupled receptors (GPCRs) (*orange*), ATPases (*black*), Bacterial Rhodopsins (*dark blue*), Enzymes (*purple*), Proteases (*dark red*) and where a protein didn’t fit in these categories, others (*brown*)

It has been said that when trying to crystallise a membrane protein in Detergents what we are actually doing is creating a crystal of Detergents contaminated with protein. Membrane proteins by definition contain large surfaces of predominantly hydrophobic residues that would ordinarily reside in the core of the lipid membrane. To purify these proteins, the researcher will need to choose a suitable Detergents to solubilise the protein in preparation for purification (Rosenbusch 1990). But which Detergents is the right one? This is often the first dilemma faced in the challenge to determine a membrane protein crystal structure. The majority of membrane protein structures deposited in the PDB have been determined using crystals grown from Detergents solubilized protein using traditional vapour diffusion experiments. In these experiments the sample being crystallised is a mixture of both protein and associated Detergents, making Detergents selection a critical parameter for growing well-ordered, well-diffracting crystals. Significant progress has recently been made in the development of novel Detergents for use in membrane protein purification and Crystallisation (Chae et al. 2010; Tao et al. 2009). However, as in 2012 the alkyl maltopyranosides account for the majority of successfully used Detergents accounting for half of all structures in the database (Fig. 5.2), followed by the alkyl Glucopyranosides (23 %), Amine Oxides (7 %) and Polyoxyethylene Glycols (7 %). Transporters still account for the majority for structures determined using alkyl maltopyranosides with 89 entries, followed by Channels with 58. Still the most successful alkyl maltopyranoside Detergents is n-dodecyl-β-D-maltopyranoside (DDM), followed by n-decyl-β-D-maltopyranoside (DM).

**Fig. 5.3:**
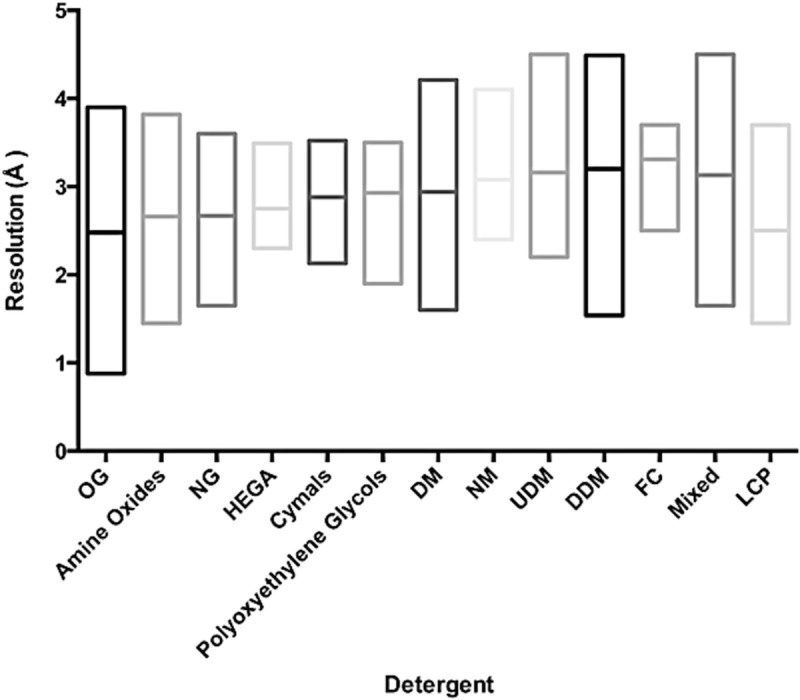
Effect of Detergents on mean resolution of reported crystal structures compared to In meso crystallisation. *Box plots* showing the lowest, highest and mean resolution (Å) reported for the most commonly used Detergents. Abbreviations are Octylglucoside (OG) (octylglucopyranoside), NG (nonlyglucopyranoside), DM (decyl maltoside), NM (nonylmaltoside), UDM (Undecyl maltoside), DDM (dodecyl maltoside), FC (fos-cholines), Mixed refers to a combination of Detergents and LCP (lipid cubic phase)

Although the choice of Detergents depends on many different parameters, considerable effort should be made to screen for crystals in shorter chain Detergents as these are more likely to diffract to a higher resolution (Sonoda et al. 2010). Analysis of the resolution of reported structures further supports this conclusion (Fig. 5.3), with the alkyl glucopyranoside Detergents, n-octyl-β-D-glucopyranoside (Octylglucoside (OG))D-glucopyranoside having both the highest resolution structure at 0.88 Å, that of a yeast aquaporin, Aqy1 (PDB: 3ZOJ) and highest mean resolution at 2.5 Å. The amine oxides, including n-lauryl dimethylamine n-oxide (Lauryldimethylamine N-oxide (LDAO)), gave the next most favourable mean resolution of 2.66 Å. There is unlikely to ever be a single panacea Detergents that can be applied to all types of membrane proteins. Nevertheless, the data support the continued use of DDM, DM, Octylglucoside (OG) and Lauryldimethylamine N-oxide (LDAO) as good first choice Detergents when screening crystallization conditions. A rational and intelligent approach should always be taken to Detergents screening for membrane proteins, which can now be accomplished easily using fluorescent-based methods early on in the structure determination process (Kawate and Gouaux 2006; Newstead et al. 2007). A notable change since 2012 has been the increased success of Detergents mixtures. Interestingly, all families, except the bacterial rhodopsin, have had at least one example where > 1 Detergents has been reported, suggesting this should be a common approach to adopt early on in the screening and optimisation process. However, as yet no trend exists that may hint at whether certain Detergents classes may be paired more successfully.

An important development in membrane protein Crystallisation over the past 3 years has been the increased use of the lipidic cubic phase (LCP) as a medium for crystal growth (Caffrey and Porter 2010; Caffrey 2009). This technological development has had an enormous impact on the G protein coupled receptors (GPCRs) field and is one of the main reasons for the increase in the number of structures from this group in the past few years (Ghosh et al. 2015). This methodology is sure to increase in use in the coming years. To date we have recorded 17 structures out of a total of 91, compared with 49 G protein coupled receptors (GPCRs) examples. As highlighted in Fig. 5.3, the mean resolution for structures determined in LCP is 2.5 Å, almost half an ångström lower than for the alkyl maltopyranoside Detergents and very close to the mean resolution obtained for n-octyl-glucopyranoside (Octylglucoside (OG)), which in many cases is too harsh for Alpha helical membrane proteins. This data adds further support to the early adoption of lipidic mesophase Lipidic mesophase crystallisation (LCP) in any structure project. More information on Detergents can be found in Chap. 10.1007/978-3-319-35072-1_2 of this book.

## Precipitants – How Do They Differ Between Membrane Proteins and Their Soluble Counterparts?

Our 2008 analysis of precipitants revealed a striking success for small MW PEGs in the Crystallisation of channels and transporters, with larger MW PEGs being more successful for respiratory complexes and membrane proteins with large hydrophilic domains (Newstead et al. 2008a). These trends have remained in the updated data set, with the notable appearance of small MW PEGs in the crystallization of the eukaryotic G protein coupled receptors (GPCRs) family. The successful concentration ranges have also been maintained, with small MW PEGs being successful at concentrations between 20 and 40 % v/v, and larger MW PEGs being used at lower concentrations, between 5 and 20 %. The successful use of organic molecules, such as MPD is still low, further confirming their unsuitability in general Crystallisation conditions for Alpha helical MPs, a situation that is dramatically different for outer membrane proteins where organic molecules are clearly more successful (Newstead et al. 2008b). Of note is the absence of high salt conditions in our database. This contrasts with a recent analysis of Crystallisation space reported for the entire PDB in 2014 (Fazio et al. 2015). This analysis clearly demonstrates the most successful crystallization reagents are PEG 3350 and ammonium sulphate, which only make up 4.0 and 3.5 % of our database, respectively. This contrasts with PEG 400, which accounts for 33 % of the reported membrane protein conditions, but doesn’t appear in the ten most abundant chemicals reported in a non-redundant analysis of successful crystallisation conditions.

## MemGold and MemGold2 – What’s the Difference?

**Fig. 5.4:**
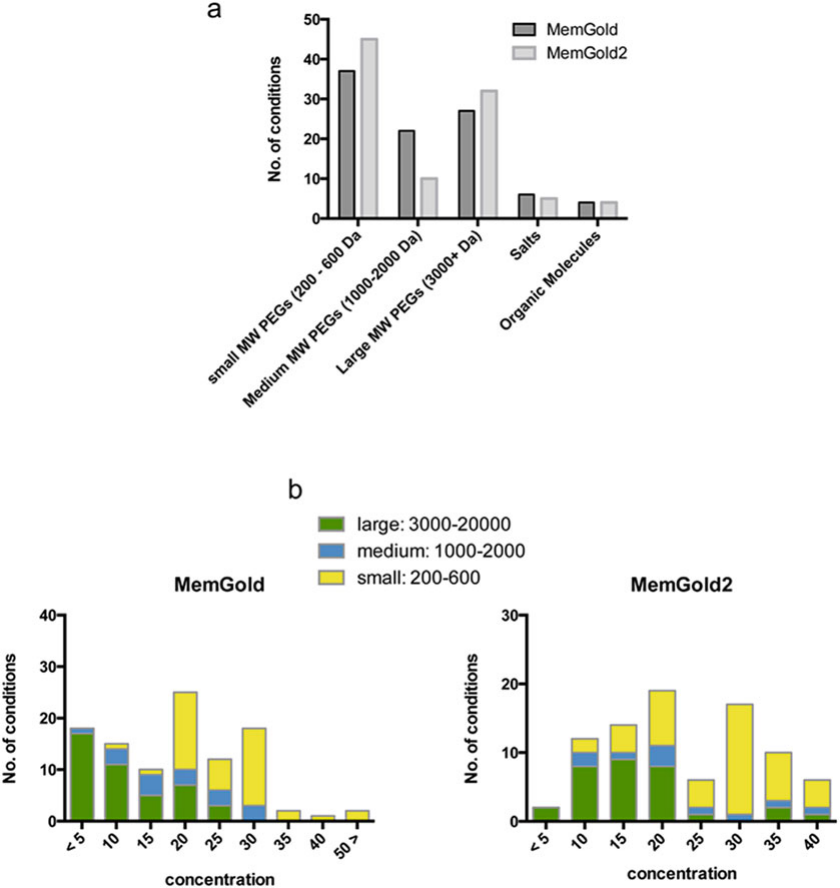
A comparison of MemGold and MemGold2 precipitant conditions. (**a**) Number of conditions that utilize PEGs, salts and organic molecules as the major precipitant solution. (**b**) Analysis of the concentration ranges of PEGs

MemGold was the first rationally designed sparse matrix style membrane protein Crystallisation screen; the previous screens developed by Jeff Abramson and So Iwata were based on a more systematic screening of PEG 400 and 4000 (Iwata 2003). MemGold was designed based on the then available 121 structures published in the PDB in 2008 and proved to be very successful as a tool for discovering initial crystallisation conditions. However, it was unclear to us at the time whether these conditions were the most optimal, given that 24 % of the conditions were contributed from the respiratory complex family. Respiratory complexes often have much larger extracellular domains that tend to dominate the crystal contacts in the unit cell, which suggested to us that perhaps these proteins tended to favour larger MW PEGs over the more hydrophobic channels and transporters. As the number of respiratory complexes has reduced relative to that of transporters and channels, we designed a new screen based on our 2012 analyses, MemGold2 (Parker and Newstead 2012). As can be seen from Fig. 5.4a, there is a noticeable difference in Crystallisation precipitants between MemGold and MemGold2. In particular, we observe an increase in the number of small (200–600 Da) and large MW PEGs (>3000 Da) with a decrease in the medium MW PEGs (1000–2000 Da). The two screensalso differ with respect to the concentration ranges of the precipitants (Fig. 5.4b), reflecting the differences in the make up of the database. In particular, the concentration ranges of all the PEGs have shifted to higher values, with large MW PEGs around 15–20 % (w/v), medium MW PEGs evenly distributed between 10 and 40 % (w/v) and small MW PEGs clustering around the 30 % (v/v) range.

## Buffers, pH and Salts

**Fig. 5.5:**
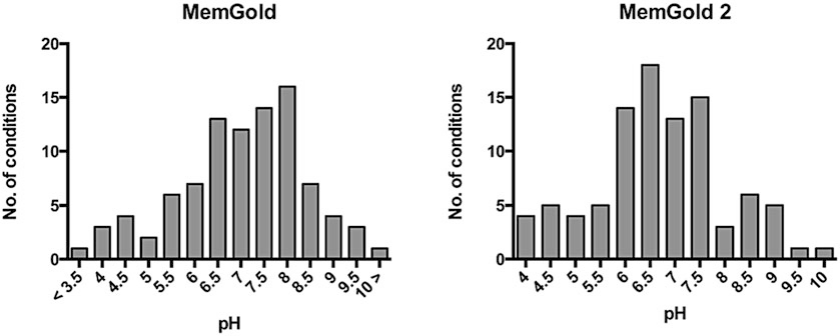
Analysis of the pH ranges screened in MemGold and MemGold2

Buffering chemicals and salts often have a significant impact on protein Crystallisation; in particular polyvalent cations and anions are often essential for crystallisation (Newman 2004; Trakhanov and Quiocho 1995). In MemGold we observed an equal split between pH 7 and pH 8, which were the most successful pH values reported (Fig. 5.5). In MemGold2 however we noticed the number of pH 8 conditions reduced markedly, with pH 6 and 6.5 increasing. This suggests that pH range is an important parameter to optimize and consider when designing membrane protein screens. We also noticed that the spread of pH values from 3.0 to 10.5 appears to be wider for membrane proteins than the recent analysis of the entire PDB, which is fairly narrow between 5 and 9 (Fazio et al. 2015).

We have also observed a significant increase in the number of different polyvalent cations and anions reported. Therefore MemGold2 contains a different set of these chemicals, which can be essential to enable proteins to Protein interactions and pack into a crystal (PepT_St_) (Solcan et al. 2012). It is interesting to note that one of the most successful commercial Crystallisation screens is the Hampton PEG/Ion screen, which also involves screening many different polyvalent and monovalent salts against the most successful precipitant for soluble proteins, PEG 3350 (Fazio et al. 2015). This is possibly something that should be replicated for membrane proteins.

## MemAdvantage – An Alpha helical Membrane Protein Additive Screen

**Fig. 5.6:**
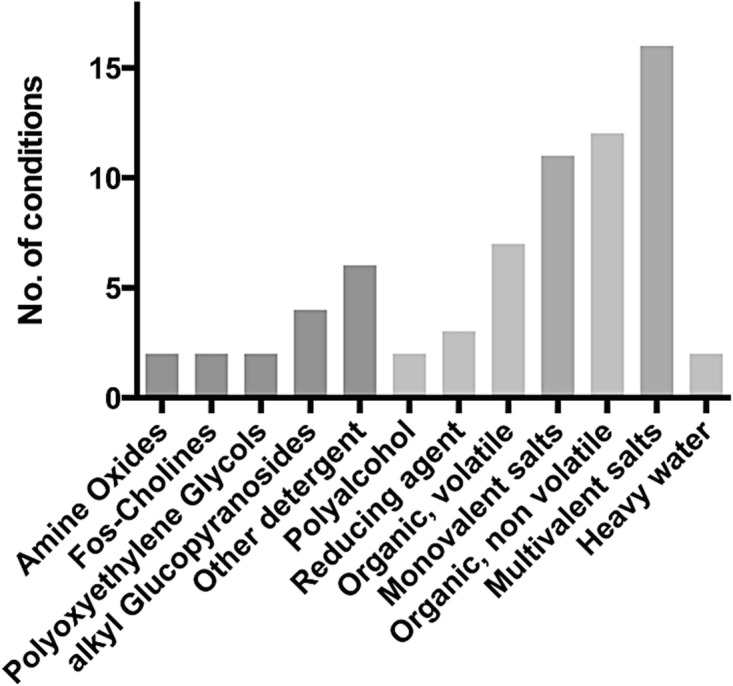
Breakdown of additives used in MemAdvantage screen for crystal Crystal optimisation

For many projects, an initial crystal condition will require optimisation, the addition of small molecules, salts, and specific ligands are well-established methods in this regard (McPherson and Cudney 2014; Luft et al. 2007). Figure 5.6 shows the range of different small molecule and salt additives that have been reported to improve initial Crystallisation conditions for Alpha helical MPs (Parker and Newstead 2012). As observed previously, mono- and multivalent salts appear prominently in the database. This no doubt due to the role these ions play in mediating intermolecular contacts during crystallisation. A notable difference however is a substantial increase in the number of secondary Detergents and non-volatile organic molecules that are now being recorded. Structures of transporters account for much of this increase, suggesting screening secondary Detergents for members of this family would be especially worthwhile. Interestingly, the reported use of additional lipids as additives appears to be mainly isolated to channels, with monovalent salts being more successful for transporters. It is clear that improving the initial Crystallisation hits can be achieved using secondary additives. MemAdvantage was designed to facilitate membrane protein Crystal optimisation by providing a convenient 96 well format to screen initial crystal conditions against the most successful additives reported in the PDB. However, given the growth of membrane protein structures, further development in this area is likely to continue, especially given the currently small number of lipids, which have shown promise in recent years as a way to improve crystal Alpha helical (Gourdon et al. 2011; Malinauskaite et al. 2014).

## MemMeso – A Systematically Designed In meso crystallisation Screen

**Fig. 5.7:**
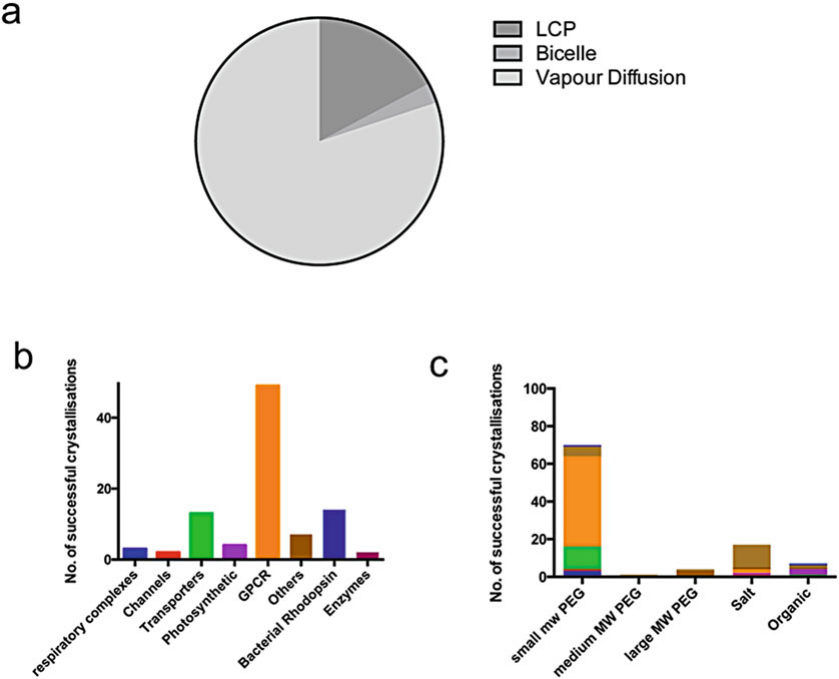
Current trends in *in meso*In meso crystallisation of Alpha helical membrane proteins. (**a**) Pie chart showing the proportion of structures within our database crystallised using either vapour diffusion, LCP or bicelle methods. (**b**) Breakdown of the LCP crystal structures into different family classes as shown in Fig. 5.1. (**c**) Analysis of types of precipitants used in LCP

The recent success of In meso crystallisation has prompted the question of whether the current commercial screening kits are suitable for this methodology. To date there are > 90 structures of membrane proteins solved using this method (Fig. 5.7a). 53 % of these are G protein coupled receptors (GPCRs) with the next most successful class of protein being transporters, at 18 % (Fig. 5.7b). However, considering that over half the examples are G protein coupled receptors (GPCRs) that were crystallised using protein fused to T4 lysozyme or apocytochrome b(562)RIL (BRIL), it would be premature to attempt a rational analysis that could be extended more generally at this time. However, if your area of research is G protein coupled receptors (GPCRs) structural biology the current examples would seem a productive starting point for further screen design. That being said, Molecular Dimensions Ltd. recently released a systematically designed *in meso* crystallization screen, MemMeso, based on work carried out in the laboratory of Osamu Nureki in the University of Tokyo, Japan. This screen comprises only small MW PEGs (200–600), four pH conditions (5, 6, 7 and 8) and 9 different salt conditions.

Our database contains 91 unique examples of membrane proteins crystallised using the *in meso*LCP method. The CrystallisationIn meso crystallisation conditions from these examples have been analysed (Fig. 5.7c). As expected, the conditions are dominated by PEG 400. However, this result is heavily biased by the G protein coupled receptors (GPCRs) examples. Interestingly we observe a number of conditions using small organic molecules, which we had previously observed were largely unsuccessful for vapour diffusion crystallisation of membrane proteins (Newstead et al. 2008a). For example the Ca^2+^/H^+^ antiporter (PDB: 4KPP) was crystallised using pentaerythritol propoxylate and sensory rhodopsin I (PDB: 1XIO) was crystallised using MPD. The remaining examples in the organics are Jeffamine-M600, which is similar in chemical composition to polyethylene glycol. Interestingly we also observe a significant number of high salt conditions, contributed by Bacteriorhodopsin, Halorhodosin and sensory rhodopsin II. Although the number of examples in our analysis are small, it suggests that crystallisation in the lipidic cubic phase may be influenced differently to that in solution.

## Conclusions

Membrane proteins represent important pharmaceutical targets and interesting subjects of study with respect to cellular biology and protein biochemistry. However, they still represent challenging targets to crystallise and study. To date our database of 569 unique structures compares to > 110,000 structures in the entire PDB, representing < 1 % of known crystal structures. The field of membrane protein structural biology is still developing at a rapid pace. The introduction of serial injection systems for crystals at synchrotron radiation and free electron sources (Conrad et al. 2015) and the development of *in situ* diffraction data collection methodology (Huang et al. 2015) suggest that what structural biologists need from a Crystallisation experiment is likely to change in the coming years. The final chapter on the topic of crystal screen design and optimization is far from being written. As more information is gathered it seems likely that new trends will be discovered and new Crystallisation methods invented or traditional methods refined to meet the growing need to understand these important and fascinating proteins at atomic resolution. The information contained in this chapter represents the current snapshot of ‘crystallisation space’ for Alpha helical membrane proteins. It is our wish that this information will encourage the efficient use of the MemGold family of screens but also enable the design of more tailored crystallisation screens for particular projects of interest to you.
